# DNA-binding directs the localization of a membrane-integrated receptor of the ToxR family

**DOI:** 10.1038/s42003-018-0248-7

**Published:** 2019-01-04

**Authors:** Sophie Brameyer, Thomas C. Rösch, Jihad El Andari, Elisabeth Hoyer, Julia Schwarz, Peter L. Graumann, Kirsten Jung

**Affiliations:** 1 Munich Center for Integrated Protein Science (CiPSM) at the Department of Biology I, Microbiology, Ludwig-Maximilians-Universität München, Martinsried, Germany; 2 LOEWE SYNMIKRO, LOEWE Center for Synthetic Microbiology and Department of Chemistry, Philipps-Universität Marburg, Hans-Meerwein Strasse, Marburg, Germany

## Abstract

All living cells have a large number of proteins that are anchored with one transmembrane helix in the cytoplasmic membrane. Almost nothing is known about their spatiotemporal organization in whole cells. Here we report on the localization and dynamics of one representative, the pH sensor and transcriptional regulator CadC in *Escherichia coli*. Fluorophore-tagged CadC was detectable as distinct cluster only when the receptor was activated by external stress, which results in DNA-binding. Clusters immediately disappeared under non-stress conditions. CadC variants that mimic the active state of CadC independent of environmental stimuli corroborated the correlation between CadC clustering and binding to the DNA, as did altering the number or location of the DNA-binding site(s) in whole cells. These studies reveal a novel diffusion-and-capture mechanism to organize a membrane-integrated receptor dependent on the DNA in a rod-shaped bacterium.

## Abstract (web summary)

Brameyer et al. identify a diffusion-and-capture mechanism responsible for the localization of the membrane integrated receptor and transcriptional activator CadC in *E. coli*. They find that external stress activates CadC, resulting in DNA-binding and cluster formation in the cytoplasmic membrane.

## Introduction

Proteins anchored with a single transmembrane helix in the cytoplasmic membrane represent the most abundant and functionally diverse category of membrane proteins in humans, in Arabidopsis, as well as in bacteria and archaea^1^. These proteins participate in many cellular functions, such as regulation of signaling, transport, and metabolism or cell division^1^. For example, recent analysis indicate *Escherichia coli* K-12 and *Vibrio harveyi* ATCC116 contain 164 and 340 proteins, respectively, that fall into this category (bioinformatics analysis by Marina Parr, unpublished), which accounts for about 40% of the bacterium’s membrane proteome. Among these proteins are the members of the ToxR receptor family. These low-abundance receptors (<100 molecules per cell) are in stark contrast to about 10,000 chemoreceptor molecules arranged in “trimer of dimers” complexes at or near the cell pole, where they allow signal integration, amplification, and adaptation during chemotactic responses^2^.

ToxR receptors combine sensory and output function within one polypeptide, and signal transduction is mediated without chemical modification^3^. These receptors are characterized by a modular structure: a periplasmic sensory domain followed by a single transmembrane helix, which is connected via a linker to a cytoplasmic DNA-binding domain^4^. Members of this receptor family include the main regulator for virulence ToxR in *Vibrio cholerae*, TcpP and TfoS in *V. cholerae*^5^, PsaE in *Yersinia tuberculosis*^6^, WmpR in *Pseudoalteromonas tunicata*^7^, and the pH-stress-sensing receptor CadC in *E. coli* and *Vibrio* species^8^. Owing to their membrane-anchoring, these transcriptional regulators are limited in their spatial dynamics, which raises the important question about their spatiotemporal localization in their active and inactive states.

**Fig. 1:**
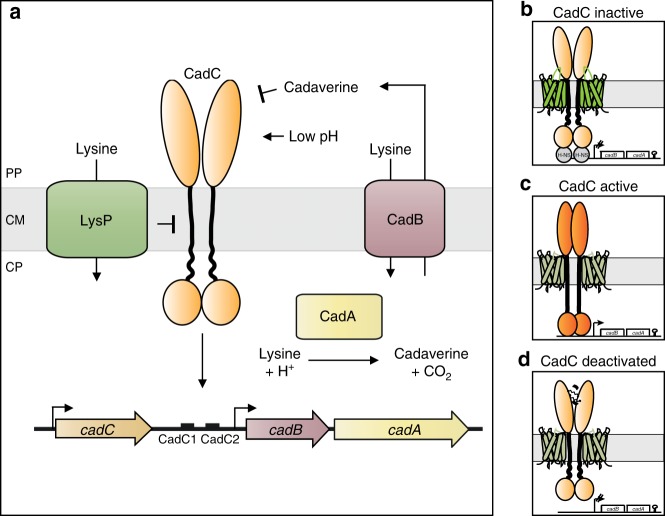
Cad-system of *E. coli*. **a** Under acid stress in a lysine-rich environment, CadC activates expression of the *cadBA* operon, encoding the lysine decarboxylase CadA and the lysine/cadaverine antiporter CadB. CadA converts lysine under consumption of a proton into the more alkaline cadaverine and carbon dioxide, thus raising the intracellular pH. The antiporter CadB transports lysine into the cells and exports cadaverine. The more alkaline cadaverine increases the extracellular pH. The availability of external lysine is transduced to CadC by the co-sensor and inhibitor LysP, a lysine-specific transporter. Moreover, external cadaverine has a feedback inhibitory effect on CadC activity. The *cadC* gene is located upstream of the *cadBA* operon and there are two CadC-binding sites (CadC1, CadC2) within the *cadBA* promoter. Each binding site is occupied by one CadC homodimer. Illustration of the three states of CadC on the right side: **b** CadC and LysP are inactive at physiological pH in the absence of external lysine; H-NS binds to the promoter; **c** CadC is activated by low pH, and LysP is activated by lysine; **d** CadC is deactivated by cadaverine. PP periplasm, CM cytoplasmic membrane, CP cytoplasm

In this study, we are focusing on the subcellular localization and dynamics of the acid stress-responsive regulator CadC in *E. coli*. Under acidic stress in a lysine-rich environment, CadC activates the expression of the *cadBA* operon, coding for the lysine decarboxylase CadA and the lysine/cadaverine antiporter CadB^9^ (Fig. 1). The pH-sensory function as well as the feedback inhibition by cadaverine could be assigned to distinct amino acids within the periplasmic sensory domain of CadC^10,11^. We find that the availability of external lysine is transduced to CadC via the co-sensor and inhibitor LysP, a lysine-specific transporter^12,13^. CadC dimerization is inhibited by LysP via intramembrane and periplasmic contacts under non-inducing conditions^12,13^. A drop in external pH induces dimerization of the periplasmic sensory domain of CadC followed by structural rearrangement of its cytoplasmic linker^10,14,15^. This permits the DNA-binding domain to homodimerize and enables *cadBA* expression^8^ (Fig. 1).

The molecular mechanism of stimulus perception and signaling by CadC is already well understood^16^. Here we are able to visualize the recruitment of CadC to DNA in whole cells. Under activating (low pH and lysine) conditions, CadC appeared as distinct cluster(s). Cluster formation strongly correlated with CadC conformational states that result in DNA-binding. This study reveals a diffusion-and-capture mechanism to organize low copy number membrane-anchored receptors dependent on the DNA, while other models of receptor localization are excluded experimentally.

## Results

### Localization of the membrane-integrated pH-sensor CadC

To visualize the localization of CadC in single cells, we generated a fluorescent CadC hybrid protein. Therefore, mCherry was connected with a linker of 22 amino acids to the N-terminal DNA-binding domain of CadC. As CadC is found in only 1–3 molecules per cell^17^, expression of mCherry-*cadC* was set under control of the *tac*-promoter in the absence of the T7-polymerase (pET-mCherry-*cadC*) to slightly increase the copy number of CadC (~3–5 molecules per cell)^17^. Cells expressing this mCherry-CadC hybrid responded to external stimuli like the wild type did (Supplementary Figure 1).

**Fig. 2:**
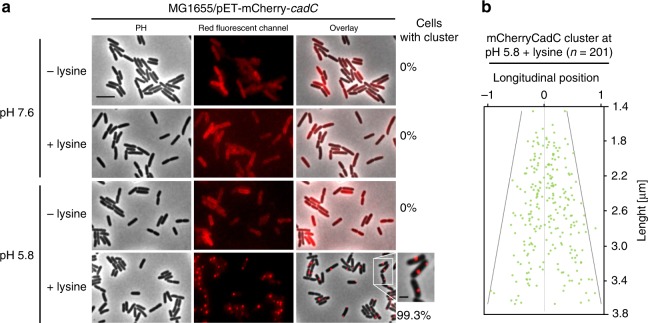
Spatio-temporal localization of mCherry-CadC under stress and non-stress conditions. **a** Fluorescent microscopic images of *E. coli*/pET-mCherry-*cadC* (3–5 molecules CadC per cell)^17^ cells grown in minimal medium (glucose as carbon source) buffered at pH 7.6 or pH 5.8, each supplemented with lysine. Images were taken 60 min after exposure to the different conditions. Numbers at the right side of the images indicate the percentage of cells with mCherry-CadC cluster (on average 450 cells were examined). PH phase contrast, scale bar = 5 µm. Magnified cropped subset of cells at pH 5.8+lysine on the right hand side, scale bar = 2 µm. **b** Position of mCherry-CadC cluster along the longitudinal axis per cell length at low pH and in the presence of lysine. Using ImageJ and the MicrobeJ plug-in, the proportion and relative position was determined. Longitudinal position: −1 and 1 =  cell pole along grey line, 0 = cell middle, *n* = 201 cells

mCherry-tagged CadC was found to be randomly distributed in the membrane of non-stressed *E. coli* cells (Fig. 2a, pH 7.6; Supplementary Figure 2) but became visible as distinct cluster(s) (1–2) under CadC-activating conditions (Fig. 2a, pH 5.8+lysine). It is important to note that cluster formation was only seen when cells were exposed simultaneously to the two stimuli (low pH and exogenous lysine) known to be required for CadC activation and induction of *cadBA*. A majority of cells contained one cluster, which was randomly distributed along the longitudinal axis of the cell under these stress conditions (Fig. 2b). In all, 16% of cells contained two mCherry-CadC clusters under this condition. As control, we determined the localization of free mCherry, which was uniformly distributed in the cytoplasm and did not form cluster under stress or non-stress conditions (Supplementary Figures 2 and 3).

**Fig. 3:**
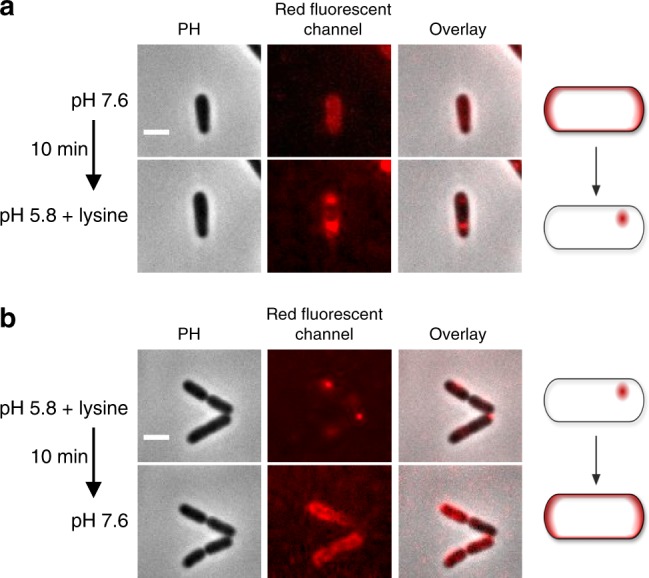
Cluster formation of mCherry-CadC is reversible. Fluorescent microscopic images of *E. coli* MG1655/pET-mCherry-*cadC* cells grown in a microfluidic growth chamber. **a** Cells were initially grown at pH 7.6 and then the medium flow was switched to stress conditions (pH 5.8+lysine). **b** Cells were initially grown at pH 5.8+lysine and then the medium flow was switched to non-stress conditions (pH 7.6). PH phase contrast, scale bar = 2 µm

Since only acid stress in a lysine-rich environment promoted cluster formation of mCherry-CadC in *E. coli*, we were interested in the reversibility of this process. To adjust the external environment and simultaneously image the location of mCherry-CadC in *E. coli* over time, we used a microfluidic growth chamber. First, we incubated cells under non-stress conditions (pH 7.6) and observed an even distribution of mCherry-CadC in the membrane (Fig. 3a). Next, we exposed cells to a medium with low pH and lysine and saw distinct clusters appearing in the cells within 10 min. Conversely, when cells were initially exposed to pH 5.8+lysine and then the medium was exchanged to non-stressed conditions (pH 7.6), the mCherry-CadC clusters disappeared within 10 min (Fig. 3b). Hence, we concluded that cluster formation of mCherry-CadC is reversible within minutes and depends on external conditions.

### Environmental stimuli trigger CadC cluster formation

As cluster formation of mCherry-CadC occurred specifically at low pH in a lysine-rich environment (Fig. 2a), we investigated the effect of environmental parameters on cluster formation in more detail. The *cadBA* operon is activated by CadC at low pH (<pH 6.8) and in the presence of lysine (>1 mM)^9^. There is feed-back inhibition by the end product of lysine decarboxylation, cadaverine (IC_50_ value of 1 mM)^11,18^ (Fig. 1).

**Fig. 4:**
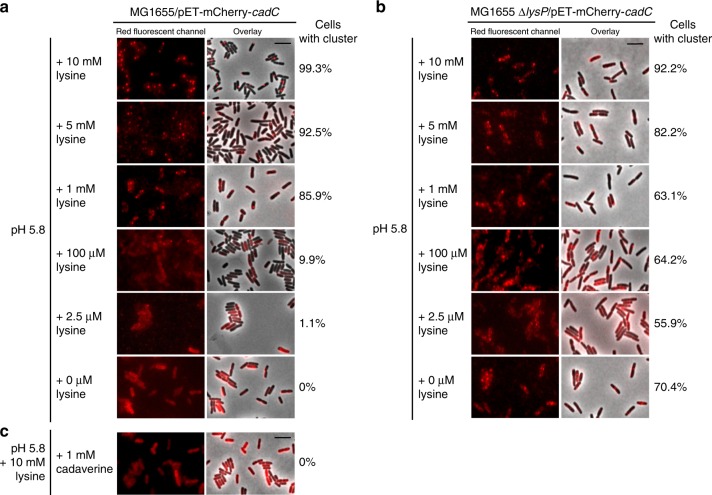
Influence of external lysine, cadaverine, and the co-sensor LysP on mCherry-CadC cluster formation. Fluorescent microscopic images of *E. coli* **a** MG1655 and **b** MG1655Δ*lysP* cells producing mCherry-CadC grown in minimal medium buffered at pH 5.8 and supplemented with different lysine concentrations for 60 min. **c** *E. coli* MG1655 cells producing mCherry-CadC grown in minimal medium buffered at pH 5.8 and supplemented with lysine and 1 mM cadaverine for 60 min. Numbers at the right side of the images indicate the percentage of cells with mCherry-CadC cluster (on average 450 cells were examined). Scale bar = 5 µm

We observed a concentration-dependent effect of external lysine at constant low pH not only for *cadBA* expression (Supplementary Figure 4) but also for mCherry-CadC cluster formation (Fig. 4a). The number of cells with a mCherry-CadC cluster greatly decreased when the lysine concentration dropped below the threshold of 1 mM. At lower lysine concentrations, e.g. (100 µM), only 13% of cells contained a mCherry-CadC cluster. At even lower concentrations, there was no cluster formation (Fig. 4a, Supplementary Table 1).

The lysine transporter LysP functions as a co-sensor for lysine in the Cad-system; in the absence of lysine, it inhibits CadC activity^12^ (Fig. 1). A *lysP* mutant strain allows *cadBA* expression in the absence of lysine^12^ (Supplementary Figure 4). Accordingly, cluster formation of mCherry-CadC in a ∆*lysP* mutant became independent of the external lysine concentrations and could even be detected in the absence of lysine (Fig. 4b).

Besides lysine and low pH, CadC activity is also influenced by cadaverine^11,18^. As expected, adding cadaverine in millimolar concentration to the medium (1 mM) prevented mCherry-CadC cluster formation (Fig. 4c, Supplementary Table 1).

### Spatio-temporal localization of CadC variants

**Fig. 5:**
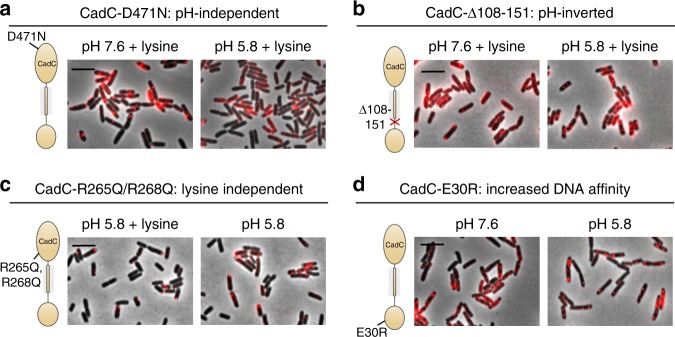
Altered stress response and cluster formation of mCherry-CadC variants. Fluorescent microscopic images of *E. coli* cells producing **a** mCherry-CadC-D471N, **b** mCherry-CadC-Δ108–151 **c** mCherry-CadC-R265Q/R268Q, and **d** mCherry-CadC-E30R. Images were taken 60 min after cells were exposed to the indicated stress conditions. Schematics mark the location of amino acid substitutions in CadC. The overlay images of phase contrast and fluorescent images are shown. Scale bar = 5 µm

Our results thus far suggest that cluster formation strongly correlates with the active state of CadC that results in DNA-binding. To confirm this finding, we next focused on CadC variants, which cause an environmental stress-independent ON-phenotype due to distinct amino acid replacements. First, we investigated the subcellular localization of pH-independent variant CadC-D471N, which results in an ON-state at low and neutral pH^10^ (Supplementary Figure 5). mCherry-CadC-D471N formed clusters independent of the surrounding pH (pH 7.6 and pH 5.8, respectively); however, it required the presence of lysine (Fig. 5a; Supplementary Table 1).

Variant CadC-∆108–151, which lacks the linker, dimerizes preferentially at physiological pH and is characterized by a deregulated *cadBA* expression profile^8^ (Supplementary Figure 5). This altered pH response can be explained by the correct perception of the external stimuli by the periplasmic domain, which are, however, incorrectly interpreted at the cytoplasmic side due to the missing linker. Consequently, clusters of this variant were observed at pH 7.6 and pH 5.8 (Fig. 5b; Supplementary Table 1). Concurrently, these results reveal that cluster formation is not a result of a lower cytoplasmic pH in *E. coli* under acid stress, which might also affect the structure of the chromosome^19^.

Information about external lysine is transduced by LysP to CadC via conformational changes within the heterooligomeric interaction. For this interaction, polar residues like R265 and R268 in the periplasmic domain of CadC are known to stabilize the interaction with LysP by a salt bridge. Substitution of these two residues leads to lysine-independent *cadBA* promoter activation at low pH^13^ (Supplementary Figure 5). Accordingly, the lysine-independent variant mCherry-CadC-R265Q/R268Q formed clusters at low pH in the presence or absence of lysine (Fig. 5c; Supplementary Table 1).

Finally, the amino acid substitution E30R within the DNA-binding domain of CadC suffices to increase the affinity of CadC to its binding site by providing additional contacts to the DNA due to the introduction of the positive charge of arginine^20^. The corresponding CadC variant causes constitutive *cadBA* expression (Supplementary Figure 5)^20^. Owing to its increased DNA-binding affinity, cluster formation became independent of pH and lysine as stimuli. Moreover, we observed multiple clusters revealing unspecific binding of CadC to the DNA (Fig. 5d; Supplementary Table 1).

### DNA directs the localization of CadC

**Fig. 6:**
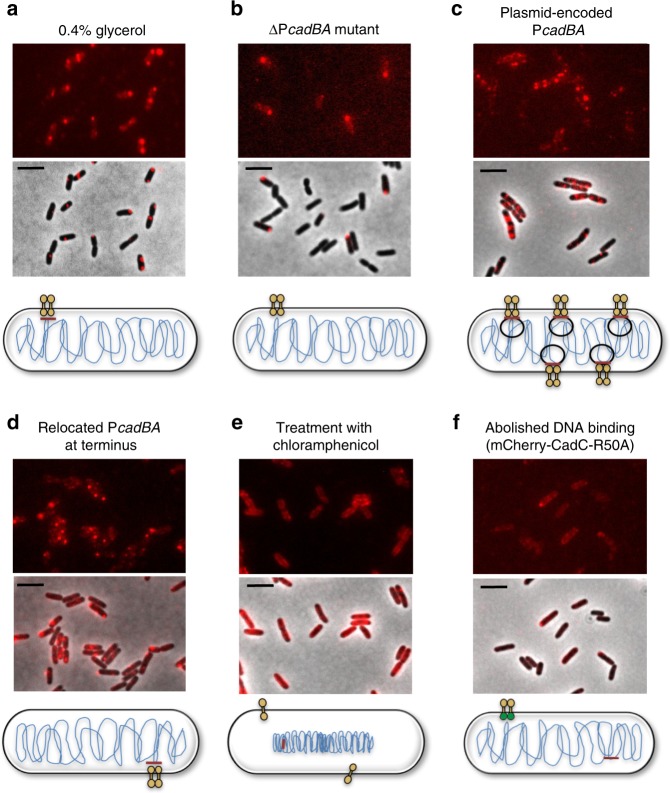
The number of CadC-binding sites influences cluster formation of mCherry-CadC. **a** Fluorescent microscopic images of *E. coli*/pET-mCherry-*cadC* (3–5 molecules CadC per cell) after cultivation in minimal medium (glycerol as carbon source) under stress conditions (pH 5.8+lysine). **b** Fluorescent microscopic images of *E. coli* MG1655Δ*cadBA* cells producing mCherry-CadC after cultivation in minimal medium (glucose as carbon source) (pH 5.8+lysine). **c** Fluorescent microscopic images of *E. coli*/pET-mCherry-*cadC* after co-transformation with plasmid pBBR1-MCS5-P_*cadBA*_-*lux* under stress conditions, cultivation as in **b**. **d** Fluorescent microscopic images of *E. coli* MG1655ΔP_*cadBA*_*_*P_*cadBA*__terminus producing mCherry-CadC under stress conditions, cultivation as in **b**. **e** Fluorescent microscopic images of *E. coli*/pET-mCherry-*cadC* after treatment with chloramphenicol in minimal medium pH 5.8+lysine (glucose as carbon source). **f** Fluorescent microscopic images of *E. coli*/pET-mCherry-*cadC*-R50A after cultivation in minimal medium, cultivation as in **b**. All images were taken 60 min after exposure to the different conditions. Schematics illustrate alterations of the CadC-binding site(s) in whole cells and CadC-R50A variant is marked with a green DNA-binding domain. Scale bar = 5 µm

CadC is a transcriptional regulator, which binds after activation to its binding site within the *cadBA* promoter. In our next experiments, we addressed the question of whether the number of CadC-binding sites correlates with the number of clusters. In fast-growing *E. coli*, bidirectional replication starts from *oriC* creating a gradient of gene dosage from the origin to the terminus^21^. If the time for replication exceeds one generation time, a new round of replication is already initiated before the previous round is completed. Thus initiation occurs at two origins in the mother cell^22–24^. Because the *cadCBA* regulon is located close to the origin (*oriC* 84 min, *cadBA* promoter 93 min^25^) in *E. coli*, the number of the CadC-binding sites can be altered by controlling the doubling time of cells. In our previous experiments, we grew *E. coli* with glucose as C source and found 16% of cells with 2 clusters (Fig. 2a). When we reduced the doubling time by growing *E. coli* with glycerol as C source at lower temperature (30 °C instead of 37 °C), we found almost exclusively (95%) only one cluster per cell (Fig. 6a).

In addition, in a mutant lacking the CadC-binding site, most cells (81%) did not form a CadC cluster, even under CadC-activating conditions (Fig. 6b). If the number of CadC-binding sites was increased by introducing the plasmid-encoded *cadBA* promoter region, multiple mCherry-CadC clusters formed within the cells (Fig. 6c). As demonstrated below, even in wild type cells only a fraction of CadC binds to DNA (Table 1); the other still freely diffusing molecules could therefore bind when multiple binding sites are available. Moreover, CadC is still capable of forming clusters even in a mutant in which the CadC-binding site has been moved to another position within the chromosome (Fig. 6d). Similarly, altering the position of *cadC* within the chromosome does not influence CadA activity (Supplementary Figure 6). Furthermore, chromosome condensation in the middle of the cell, caused by treatment with chloramphenicol^26–28^, prevents CadC cluster formation (Fig. 6e). Finally, the amino acid substitution R50A within the DNA-binding domain of CadC almost abolishes DNA-binding as recruitment and activation of the RNA polymerase on the *cadBA* promoter is inhibited^20^. This CadC variant fails to activate *cadBA* expression (Supplementary Figure 5). Likewise, cluster formation of the mCherry-tagged CadC-R50A variant was greatly reduced and only detectable in 8% of the cells (Fig. 6f; Supplementary Table 1).

**Table 1:** Influence of environmental stress on dwell times of mNeonGreen-CadC in MG1655 wild type

Condition	Short dwell time (0.05 s)	Long dwell time (0.19–0.62 s)
pH 5.8+10 mM lysine	74%	26%
pH 7.6	100%	0%

Dwell time is the time one mNG-CadC molecule spends in a radius of 250 nm (2.5 pixels). A two-component fit for mNG-CadC was assumed

### Single-molecule tracking of mNeonGreen-CadC

We tagged CadC with mNeonGreen (mNG) to investigate the dynamics of single CadC molecules during external pH changes. mNG is the brightest monomeric fluorescent protein found to date and is an excellent fusion tag for traditional imaging as well as stochastic single-molecule super-resolution imaging^29^. We therefore integrated mNG-*cadC* chromosomally in MG1655 wild type and found it to be a functional hybrid protein allowing stimulus perception and signal transduction like the wild type receptor (Supplementary Figure 7). Then we analyzed the dynamic behavior of mNG-CadC under non-stress (pH 7.6) and stress (pH 5.8+lysine) conditions in *E. coli*MG1655.

**Fig. 7:**
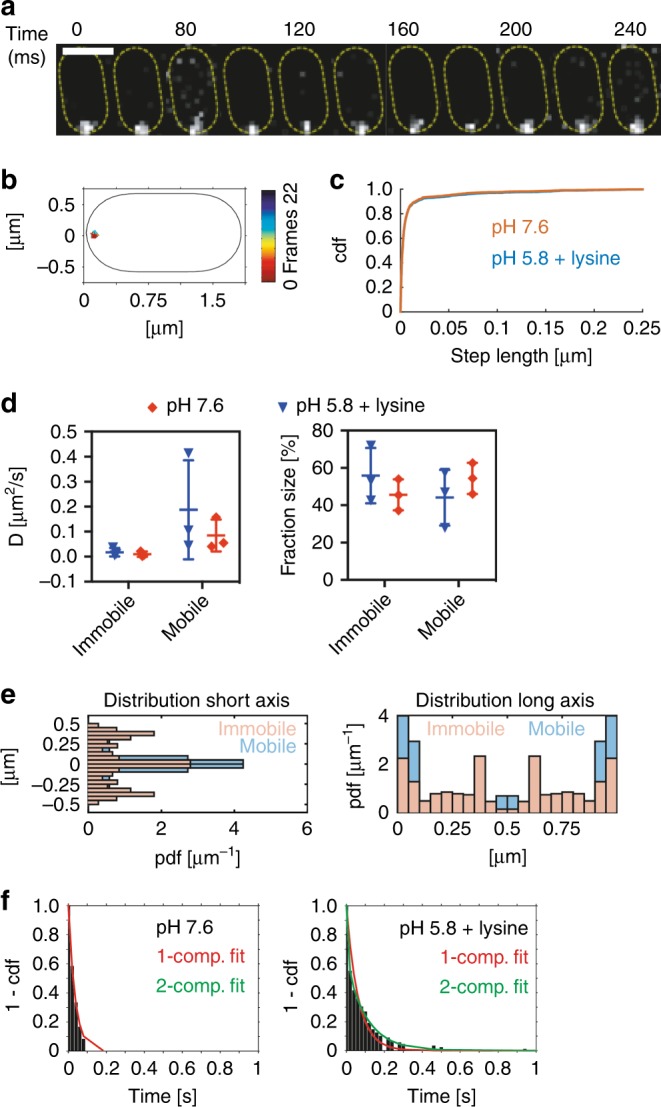
Single-molecule imaging and tracking of mNeonGreen-CadC molecules under different conditions. **a** Representative example of a single-molecule track of mNG-CadC under stress conditions in *E. coli* MG1655 wild type. Images were acquired at a frame rate of 50 Hz and montage shows track in increments of 40 ms corresponding to two time frames. Scale bar = 1 µm. **b** Trajectory of the track shown in **a**. Trajectory was color coded according to its length. **c** Cumulative probability distribution (cdf) of single step displacements. **d** Apparent diffusion coefficients (*D*, left side) and fraction size of mobile and immobile populations (right side) determined from distributions shown in **c**. **e** Representative probability distribution function (pdf) of mobile and immobile molecules under stress conditions within a standardized cell along the short (left side) and long cell axis (right side). **f** Cumulative distribution of residence times under non-stress (left side) and stress conditions (right side). Results of the 1- and 2-component fits are shown in red and green, respectively

Although the copy number of chromosomally encoded CadC is extremely low^17^, single molecules of the receptor could be localized (Fig. 7a) after a short bleaching procedure. Tracking of single molecules at a rate of 50 Hz revealed a predominant localization of the receptor close to the poles (Fig. 7b, e), which is conceivable since the CadC-binding site is close to the origin. It is important to note that single molecules of mNG-CadC were heterogeneously distributed within the population and only detectable in 50% of the cells, indicating that only half of the cells would respond to lowered pH by inducing the *cadBA* operon. We calculated the apparent diffusion constants (*D*) by analyzing the cumulative probability distribution of single frame displacements assuming that the molecules undergo mobile and immobile movement (Fig. 7c). Considering all molecules from three independent experiments (see Supplementary Table 2 for details), mNG-CadC showed an apparent diffusion rate ranging from 0.0077 to 0.018 µm^2^/s for the immobile fraction and from 0.067 to 0.189 µm^2^ /s for the mobile fraction (Fig. 7d). The latter corresponds with the diffusion rate determined for a protein with four transmembrane domains^30^. However, the diffusion rates of mNG-CadC were not affected by alterations of the external conditions. The fraction of mobile molecules was determined with about 56% (sd ± 15) under non-stress conditions and with about 46% (sd ± 8.4) under stress conditions (Fig. 7d). The distribution of all tracks projected in standardized cells from the three independent experiments (24 movies) shows that movement of mNG-CadC molecules occurs mostly at the poles and laterally at the membranes (Fig. 7e). This is consistent with the idea that CadC binds to its chromosomal-binding site within the *cadBA* promoter or diffuses randomly within the membrane.

When it came to residence time, defined as the time one mNG-CadC molecule spends in a certain radius (~120 nm), we found large differences: under non-activating conditions mNG-CadC was mostly characterized by a short dwell time (about 0.05 s). Under activating conditions, 26% mNG-CadC molecules had a long dwell time with up to 0.62 s, which underlines the idea that the DNA captures CadC and immobilizes it (Fig. 7f and Table 1).

### Different mechanisms for CadC localization

**Fig. 8:**
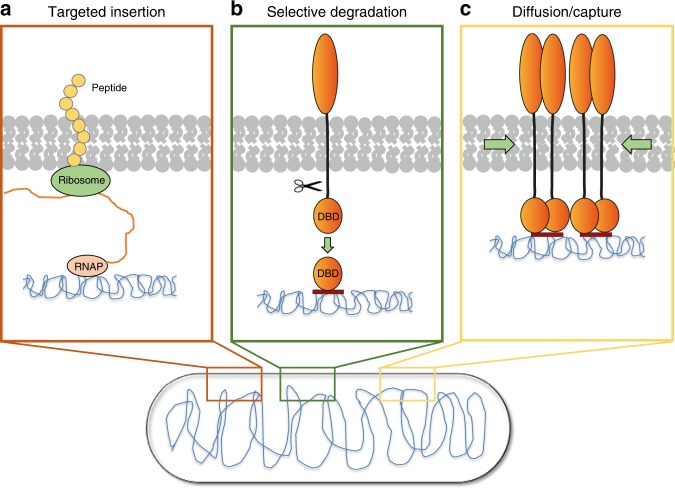
Different mechanisms for the localization of membrane-inserted proteins in bacterial cells. **a** Targeted insertion: Co-transcriptional translation with insertion of proteins directly into the membrane. **b** Selective degradation: Proteolytic cleavage of the DNA-binding domain (DBD) for subsequent transcriptional activation. **c** Diffusion/capture: Dynamic localization of proteins by diffusion driven by the interaction with DNA

Here we discuss the three models of how proteins can be localized in bacterial cells: targeted insertion, selective degradation, and diffusion/capture^31^ (Fig. 8).

With regards to targeted insertion, we could exclude the need to insert CadC into the membrane close to its binding site by generating an *E. coli* mutant, in which *cadC* was relocated to the *lac* operon. This strain showed similar CadA activity in the two stimuli-dependent manner like the wild type strain (Supplementary Figure 6).

Selective degradation is also extremely unlikely. Previous studies on the kinetics of *cadBA* expression and the deactivation of CadC by cadaverine^9,11^ rule out that CadC is proteolytically processed.

The reversibility of cluster formation of CadC (Fig. 3) as well as the correlation between the number of CadC clusters and DNA-binding sites favor the diffusion/capture model. To further confirm this model, we deleted the native CadC-binding site within the chromosome and inserted a new one at the chromosome near the terminus. Microscopic images indicated cluster formation of CadC in response to the two stimuli like the wild type did.

Furthermore, it has long been known that the treatment of *E. coli* with chloramphenicol prevents *cadBA* expression^32^. There is now substantial experimental evidence that treatment with chloramphenicol causes chromosome condensation in the middle of the cell^26–28^. Based on these results, we hypothesized that chloramphenicol does not affect the translation of one component of the Cad-system but rather prevents the physical contact between CadC and the DNA. Therefore, we treated our reporter strain with chloramphenicol and analyzed CadC cluster formation. Indeed, it prevented CadC cluster formation (Fig. 6e), and the results were similar to those for a mutant, which lacks the CadC-binding site (Fig. 6b). Taken together, these results support a localization of CadC by a diffusion/capture mechanism that is dependent on the DNA.

## Discussion

Almost nothing is known about how proteins with only one transmembrane helix, and particularly receptors and signaling proteins, are localized within cells. Besides members of the ToxR receptor family, other signaling proteins belonging to this group include the membrane-anchored cyclic di-GMP-binding protein BcsB^33^ and two-component modulators, such as SafA^34^ or MzrA^35^ in *E. coli*. With respect to the DNA-binding ToxR receptor family, the question arises whether their transcriptional activity requires a specific location within the cell.

Here we could show that the low-copy, membrane-anchored receptor CadC exhibits a dynamic localization. CadC forms distinct clusters in its active state (Figs. 2 and 7), which requires two external stimuli: low pH (<6.8) and external lysine (>1 mM). Importantly, in each case clusters formed and dissolved within minutes depending on the environmental conditions (Fig. 3). Cluster formation reflects in a perfect manner the previously determined *cadBA* expression activation pattern^9,11^. Similarly, CadC-deactivating parameters, such as neutral pH, the absence of lysine, or the presence of cadaverine not only prevent *cadBA* expression but also mCherry-CadC cluster formation (Figs. 2, 4, and 5). CadC variants with distinct amino acid replacements, which cause a stimulus-independent ON-phenotype, corroborated our conclusion that clusters of CadC indicate binding of the receptor to the DNA.

Likewise, the degree of polar subcellular localization of the sensory histidine kinases DcuS and CitA of *E. coli* is affected by their activation after binding of the corresponding ligands fumarate or citrate, respectively^36^. The histidine kinase BaeS of *E. coli* also shows a copper-induced cluster formation that happens quickly and reversibly^37^. Polar localization of the histidine kinases PleC and DivJ has been shown to control asymmetric cell division and development in *Caulobacter crescentus*^38,39^. These histidine kinases also co-localize with their regulated reaction and interaction partners, respectively. In *Pseudomonas aeruginosa*, the chemosensory-like receptor WspA is both polarly and laterally localized for correct surface sensing and cyclic-di-GMP production^40^. These examples show that, for the most part, protein–protein interactions determine the location of the corresponding receptors and that these proteins are found at the pole in the majority of cases.

Of the three models of protein localization in bacterial cells cited (Fig. 8), namely targeted insertion, selective degradation, and diffusion/capture^31^, our results revealed that CadC is localized by a diffusion/capture mechanism dependent on the DNA. The number of CadC clusters per cell correlated well with the number of available CadC-binding sites (Fig. 6). For example, cells frequently had more than one mCherry-CadC cluster in fast growing rather than in slowly growing cultures (Figs. 2a and 6a). This phenomenon can be explained by the parallel replication of several chromosomes in fast-growing cells. The number of CadC clusters was further increased in cells having the CadC-binding site on a plasmid, which artificially increased the number of potential CadC-binding sites (Fig. 6c).

The diffusion–capture mechanism is also supported by the reversibility of the process, which in turn depends on the oligomeric state of CadC. In general, diffusion of membrane-integrated proteins is 4–5 times slower than that of cytoplasmic proteins. In addition, the diffusion of membrane proteins depends heavily on their size and on the number of transmembrane helices^30^. Accordingly, we would expect a higher diffusion rate of CadC before the receptor dimerizes after activation^14^. The presence of two CadC-binding sites, Cad1 and Cad2, within the *cadBA* promoter, moreover, argues for the binding of two CadC dimers being required to discriminate between transcription in the ON and OFF states^41^. This requirement explains that binding of CadC to the DNA results in an accumulation of molecules that become visible as clusters. Single-molecule tracking of CadC indicated a splitting of the dwell times into one short and one long time when cells were under acidic stress in a lysine-rich environment, supporting the idea that a fraction of CadC is immobilized by DNA-binding under this condition (Fig. 7, Table 1). Similarly, the main regulator of virulence ToxR requires the membrane-anchored co-activator TcpP to activate target gene expression in *V. cholerae*. Localization studies of TcpP indicated its heterogeneous diffusion, with three populations being observed—one fast, one slow, and one immobile. These results suggest the recruitment of TcpP by ToxR for binding to the DNA and thus activating transcription^42^.

Finally, Kumar and colleagues^30^ found that the mobility of soluble transcription factors depends on their binding specificity: those that make less specific contacts move faster. The crystal structure of the CadC DNA-binding domain resembles OmpR effector domains^20^. Our previous structure and function analyses of the CadC–DNA complex revealed that CadC mainly employs non-sequence-specific contacts and makes only a few specific contacts with the DNA^20^. This lets the CadC molecule move within the membrane until it finds the correct binding site. This statement is supported by the finding that CadC also forms clusters in a mutant in which the binding site has been relocated to another position within the chromosome. Due to unspecific contacts, some CadC clusters were even detected in a mutant lacking the CadC-binding site^20^. Furthermore, previous work by Smartzidou and colleagues revealed that CadC has an effect on the *ompC* and *ompF* expression^43^. Although there is only low match between the CadC1-binding site in the *cadBA* promoter and the regulatory region upstream of *ompF*, it might be that CadC forms a cluster at this locus.

In conclusion, to our knowledge this is the first example of DNA directing the localization of a membrane-integrated protein. It expands the repertoire of biomolecules with signatures that contribute to subcellular organization. In future studies, we will address whether proteins like CadC also contribute to the interaction between bacterial chromosome and cytoplasmic membrane^44^.

## Methods

### Bacteria and growth conditions

Bacterial strains and plasmids used in this study are listed in Supplementary Table 3 and oligonucleotides in Supplementary Data 1. *E. coli* strains were cultivated in LB medium (10 g/l NaCl, 10 g/l tryptone, 5 g/l yeast extract) or in Kim Epstein (KE) medium^45^ adjusted to pH 5.8 or pH 7.6 using the corresponding phosphate-buffered medium and incubated aerobically in a rotary shaker at 37 °C. KE medium was always supplemented with 0.2% (w/v) glucose; however, if so stated 0.4% (w/v) glycerol was used instead, and cells were incubated aerobically in a rotary shaker at 30 °C.

If necessary, media were supplemented with 100 µg/ml ampicillin or 50 µg/ml kanamycin sulfate. To allow the growth of the conjugation strain *E. coli* WM3064, we added *meso*-diamino-pimelic acid (DAP) to a final concentration of 300 µM. Generally, lysine was added to a final concentration of 10 mM unless otherwise stated.

### In vivo fluorescence microscopy

To analyze the spatial localization of CadC and its variants, *E. coli* MG1655, *E. coli* MG1655Δ*lysP*, MG1655ΔP_*cadBA*_, or *E. coli* MG1655ΔP_*cadBA*_*_*P_*cadBA*__ terminus were transformed with plasmids encoding mCherry-tagged CadC and CadC variants by electroporation. *E. coli* strains carrying the plasmids were cultivated overnight in KE medium pH 7.6 supplemented with ampicillin. The overnight cultures were used to inoculate (OD_600_ of 0.1) in fresh KE medium pH 7.6 supplemented with ampicillin and cells were aerobically cultivated at 37 °C. At an OD_600_ of 0.5, the cells were gently centrifuged and resuspended, thereby exposing them to different conditions: KE medium pH 7.6; KE medium pH 7.6+lysine; KE medium pH 5.8 or KE medium pH 5.8+lysine, each supplemented with ampicillin. Then the cultures were aerobically cultivated at 37 °C. At certain intervals (10, 30, 60, 120, 180, and 240 min), 2 µl of the culture was spotted on 1% (w/v) agarose pads (prepared with the different media), placed onto microscopic slides, and covered with a coverslip. Subsequently, images were taken on a Leica DMi8 inverted microscope equipped with a Leica DFC365 FX camera (Wetzlar, Germany). An excitation wavelength of 546 nm and a 605-nm emission filter with a 75-nm bandwidth was used for mCherry fluorescence for 750 ms, gain 5, and 100% intensity.

To monitor the effect of chloramphenicol on the ability of CadC to form clusters, *E. coli* MG1655 was transformed with plasmid pET-mCherry-CadC by electroporation. Cells were cultivated as described above; however, before exposing cells to KE medium pH 5.8+lysine, they were incubated for 5 min with 400 µg/ml chloramphenicol.

To visualize the effect of multiple CadC-binding sites, MG1655 wild type was co-transformed with plasmids pET-mCherry-CadC and pBBR1-MCS5-P_*cadBA*_-*lux* via electroporation. This strain was cultivated in medium supplemented with ampicillin and gentamycin.

The location of *oriC* (84.3 min), the *cadCBA* regulon (93.9 min), and the terminus (33.7 min) on the chromosome of *E. coli* was determined using http://www.ecogene.org^25^.

The position of mCherry-CadC cluster along the longitudinal axis per cell length was determined using ImageJ^46^ and the MicrobeJ^47^ plug-in using default settings to identify rod-shaped bacteria and fluorescent maxima.

### Time lapse fluorescence microscopy

To visualize the temporal and spatial localization of CadC in vivo, *E. coli* MG1655/pET-mCherry-*cadC* was cultivated as described above. At an OD_600_ of 0.5–0.7, the cells were adjusted to an OD_600_ of 0.1 and 80 µl were pipetted into the microfluidic growth chamber CellAsic ONIX B04A-03 (Chromaphor, Oberhausen, Germany). This microfluidic growth chamber allows growth only in one dimension resulting in a single layer of cells; however, cells are surrounded by a constant flow of medium. Cells were grown according to the manufacturer's instructions. Briefly, cells were pipetted in the inlet well, and the microfluidic growth chamber was placed on a Leica DMi8 inverted microscope equipped with a Leica DFC365 FX camera. The plate was then covered with the manifold and sealed by turning on the vacuum pump connected to the Microfluidic Flow Control Panel ONIX. Cells were then flushed into the trapping region with a flow of 10 psi (equals 15 µl/h) for 10 s. Non-trapped cells were washed away with a flow of 4 psi (equals 5 µl/h) with KE medium for 5 min. Afterwards, a constant flow of 1 psi (equals 2 µl/h) was used for the whole experiment for approximately 2 h with a constant temperature of 37 °C by means of an incubator (PeCon, Erbach, Germany) around the DMi8 microscope. Cells were left to grow first for 1 h either under non-stress conditions (KE medium pH 7.6) or under stress conditions (KE medium pH 5.8+lysine) to adapt to the environment. Then the pH conditions were altered using the manual Microfluidic Flow Control Panel ONIX (Chromaphor, Oberhausen, Germany), which also allows control of the flow. Overall, several positions were imaged every 5 min with an excitation wavelength of 546 nm and a 605 nm emission filter with a 75 nm bandwidth for mCherry fluorescence for 750 ms, gain 5, and 100% intensity. To set the focus plane, an automatic autofocus was performed at every position and every time point using the adaptive focus control (AFC) and closed loop focus system of the DMi8 microscope. Time lapse recordings were cropped, and we selected single images before and after the pH switch of the same positions.

### Single-molecule microscopy of mNeonGreen-CadC

To analyze the spatial and temporal localization of single molecules of CadC, CadC was chromosomally tagged with mNeonGreen and visualized using a Nikon Ti-E microscope equipped with a motorized stage, a high numerical aperture objective (Nikon CFI Apochromat TIRF 100XC Oil, NA 1.49), an EM-CCD camera (ImagEM X2, Hamamatsu), and an appropriate filter set for imaging mNeonGreen molecules (YFP HC filter set; BrightLine 500/24, Beamsplitter 520, and BrightLine 542/27). For tracking individual molecules, cells were continuously illuminated with the central part of a laser beam (TOPTICA Beam Smart, 515 nm, max. power 100 mW) with an intensity of 160–320 W/cm^2^ and streams were recorded at a frame rate of 50 Hz in VisiView (Visitron Systems) for 2 min. Cells were grown as described above. At an OD_600_ of 0.5, the cells were spotted on glass cover slips (25 mm, Menzel) and covered with a 1% (w/v) agarose pad (prepared with the different media) with images taken subsequently.

Acquired movies were processed in MATLAB (Version 2014a, MathWorks) and Fiji^48^. First, we summed up all frames of a movie in Fiji to generate projected images of all movies. These projections were used to manually segment cells in Oufti^49^, and the contours served to measure the mean fluorescence intensity of all cells over time. The resulting photobleaching curve was fitted with a two-component exponential decay function to determine the time when the cells reached the single-molecule level. Tracking was then performed in u-track^50^ starting at the time point when the internal fluorescence reaches the single-molecule level. All further analysis was performed in a custom-written software (SMTracker), available upon request. Briefly, we determined the mobility of single molecules assuming a two-state model with molecules being in a free-floating state and a DNA-bound state. We applied this model to fit the step size distribution of all trajectories and estimated the diffusion rate and the size of the subpopulations according to the method suggested by Schütz et al.^51^. We calculated the dwell time by counting the time a molecule stays within a radius of defined size. The size of the radius was set by displacements of the immobile molecules. The dwell time distribution follows an exponential distribution, which can be fitted by one- and two-component decay function to determine one or two decay constants, which inversely correspond to the residence time. We only included trajectories with at least four consecutive positions in the analysis.

### Additional methods

Details of the construction of strains and plasmids, the measurement of in vivo CadC signal transduction activity, measurement of intracellular CadA activity, and Western blot experiments are provided in Supplementary Information. All primer sequences used in this study are provided in Supplementary Data 1.

## Supplementary information

Supplementary Information

Description of Additional Supplementary Files

Supplementary Data 1

## Data Availability

All relevant data are available from the authors on request.
