# Supplementary material for: DNA-binding directs the localization of a membrane-integrated receptor of the ToxR family
Source: Commun Biol. 2019 Jan 4;2:4. doi: 10.1038/s42003-018-0248-7 (PMC6320335; doi:10.1038/s42003-018-0248-7)
Supplement: Supplementary file 1 — Supplementary Information [file 42003_2018_248_MOESM1_ESM.pdf]

Supplementary Information

Material and Methods

Construction of strains and plasmids. Molecular methods were carried out according to standard protocols or according to the manufacturer's instructions. Kits for the isolation of plasmids and the purification of PCR products were purchased from Süd-Laborbedarf (SLG; Gauting, Germany). Enzymes were purchased from New England BioLabs (Frankfurt, Germany). Bacterial strains and plasmids as well as primers used in this study are summarized in Supplementary Table 3 and Supplementary Data 1, respectively.

Marker-less in-frame deletion strain MG1655ΔP$_{cadBA}$ (deletion of the *cadBA* promoter region, which includes the CadC-binding site) was constructed by Red®/ET® recombination using the *E. coli* Quick and Easy gene deletion kit (Gene Bridges, Heidelberg, Germany). Briefly, primers (PcadBA_KO_sense and PcadBA_KO_anti) were designed according to the manual. These primers target the surrounding of the promoter region of *cadBA* in order to amplify the FRT-PGK-gb2-neo-FRT template. Double stranded PCR fragment was introduced via electroporation into *E. coli* MG1655 according to the manual. Deletion of the *cadBA* promoter region was verified by colony PCR and sequencing using the primers PcadBA_check_sense and PcadBA_check_rev.

In order to gain strain *E. coli* MG1655ΔP$_{cadBA}$_P$_{cadBA}$_terminus, the *cadBA* promoter region was inserted at the terminus (33.7 minutes) in background of strain MG1655ΔP$_{cadBA}$. Briefly, the *cadBA* region was inserted between *gadC* and *gadB*. Therefore, DNA fragments comprising 650 bp of *gadC*, *gadB* and *cadBA* promoter, respectively, were amplified by PCR using MG1655 genomic DNA as template and the primer pairs gadC_OL_PcadBA_rev and PcadBA_OL_gadB_fwd, PcadBA_OL_gadB_rev and gadB_OL_pNPTS_rev, as well as pNPTS_OL_gadC_fwd and gadC_OL_PcadBA_fwd, respectively. After purification, these fragments were assembled via Gibson assembly [1] into EcoRV-digested pNPTS138-R6KT plasmid, resulting in the pNTPS138-R6KT-P$_{cadBA}$_terminus plasmid. The resulting plasmid was introduced into *E. coli* MG1655 by conjugative mating using *E. coli* WM3064 as a donor on LB medium containing DAP. Single-crossover integration mutants were selected on LB plates containing kanamycin but lacking DAP. Single colonies were then streaked out on LB plates containing 10% (wt/vol) sucrose but no NaCl to select for plasmid excision. Kanamycin-sensitive colonies were then checked for targeted insertion by colony PCR using primers

(gadC_check_fwd and gadB_check_rev) bracketing the location of the insertion and sequencing of the respective PCR fragment.

Construction of the marker-less in-frame deletion strain MG1655Δ*lysP* strain was achieved using the suicide plasmid pNPTS138-R6KT-Δ*lysP*. Briefly, DNA fragments comprising 650 bp upstream and downstream of *lysP,* respectively, were amplified by PCR using MG1655 genomic DNA as template and the primer pairs pNPTS_OL_lysP_us_fwd and us_lysP_OL_ds_rev as well as us_lysP_OL_ds_fwd and lysP_ds_OL_pNPTS_rev, respectively. After purification, these fragments were assembled via Gibson assembly [1] into EcoRV-digested pNPTS138-R6KT plasmid, resulting in the pNTPS138-R6KT-Δ*lysP* plasmid. The resulting plasmid was introduced into *E. coli* MG1655 by conjugative mating using *E. coli* WM3064 as a donor as described above. However, in order to check for targeted deletion by colony PCR, the primers LysP_check_fwd2 and LysP_check_rev2 were used bracketing the location of the deletion, followed by sequencing of the respective PCR fragment.

In order to construct plasmid pET-mCherry-*cadC*, the mCherry gene was amplified by PCR, using pBR-Cherry [2] as template and primers Cherry_NcoI_fwd and Cherry_NcoI_rev, and cloned into plasmid pET16b-*cadC* using NcoI restriction site. Correct insertion of mCherry was verified by sequencing. However, introducing a linker of 22 amino acids between mCherry and CadC (amino acid sequence: M AGH HHH HHH HHH SSG HIE GRH).

In order to tag the variants CadC-D471N, CadC-E30R, CadC-R265Q/R268Q, CadC-R50A and CadC-Δ108-151 with mCherry, plasmid pET-mCherry-*cadC* was digested with NdeI and HindIII and the backbone of 6130 bp was isolated. Simultaneously, plasmids pET-*cadC*-D471N, pET-*cadC*-E30R, pET-*cadC*-R265Q/R268Q, pET-*cadC*-R50A and pET16b-*cadC*-Δ108-151 were digested with NdeI and HindIII and the fragments of 1830 bp were isolated and cloned into the digested pET-mCherry-*cadC* plasmid. Each plasmid was verified by sequencing.

For construction of the reporter plasmid pBBR1-MCS5-P_{cadBA}-*lux*, 300 bp of the region upstream of *cadBA* was amplified by PCR using primers PcadBA_XbaI_fwd and PcadBA_XmaI_rev and MG1655 genomic DNA as template, and cloned into plasmid pBBR1-MCS5-TT-RBS-lux [3] using restriction sites XbaI and XmaI. Correct insertion was verified by sequence analysis using primer pNTPS_Seq_fwd.

For construction of the plasmid pET-mCherry, the 730 bp mCherry fragment amplified by PCR using primers fwd_mCherry_NcoI and rev_mCherry_BamHI and plasmid pBR322Cherry [2] as template and cloned into plasmid pET16b using restriction sites NcoI and BamHI. The final plasmid pET-mCherry was verified by sequencing using the primer pET16b_down.

For single molecule microscopy, an *E. coli* MG1655 strain harboring chromosomally *mNG*-tagged *cadC* under control of its native promoter was constructed. In-frame insertion of *mNG* was achieved in *E. coli* MG1655 using the suicide plasmid pNPTS138-R6KT-*mNG-cadC*. Briefly, 500 bp upstream of *cadC* was amplified by PCR using MG1655 genomic DNA as template and the primer pairs CadC_us_EcoRI_fwd and CadC_us_OL_mNG_rev. In order to amplify *mNeonGreen,* the primer CadC_us_OL_mNG_fwd and mNG_OL_ds_CadC_rev using the plasmid TCR361 [4] as template (750 bp). Moreover, using primer mNG_OL_ds_CadC_fwd and CadC_ds_PspO_rev and pET-mCherry-*cadC* as template, the second flanking region containing a short sequence of *cadC* gene and 22 codons for a linker (amino acids sequence: M AGH HHH HHH HHH SSG HIE GRH) was amplified (600 bp). Using overlap extension PCR, the three fragments were assembled via their homologous regions. The overlap PCR fragment of 1740 bp was cloned into plasmid pNPTS138-R6KT using EcoRI and PspOMI restriction sites resulting in the pNPTS-mNG-*cadC* plasmid. The resulting plasmid was introduced into *E. coli* MG1655 by conjugative mating using *E. coli* WM3064 as a donor as described above for the deletion of *lysP*, only with the exception that the primer cadC-check-fwd and cadC-check-rev were used to check kanamycin-sensitive colonies for targeted N-terminal insertion of mNeonGreen in frame with *cadC*.

Measurement of CadC signal transduction activity in vivo. *In vivo* signal transduction activity of mCherry-CadC wild type and different variants was probed with a luminescence-based reporter. Their ability to activate the P_{cadBA} promoter, which controls the *luxCDABE* expression, was tested under stress and non-stress conditions using luminescence as readout. Consequently, *E. coli* MG1655 carrying pET-mCherry-*cadC* or variants were co-transformed with the reporter plasmid pBBR1-MCS5-P_{cadBA}-*lux*. These strains were incubated in KE medium pH 7.6 supplemented with the respective antibiotics overnight. As controls, *E. coli* MG1655Δ*cadC* carrying plasmids pET16b-*cadC* and pBBR1-MCS5-TT-RBS-lux (promoter-less) and pET16b (empty plasmid) and pBBR1-MCS5-P_{cadBA}-*lux*, respectively, were used and cultivated in KE medium pH 7.6 supplemented with the respective antibiotics overnight. The overnight cultures were adjusted to an OD_{600} of 0.1 and were then aerobically cultivated in 96-well plates at 37 °C under four different conditions (KE medium pH 7.6; KE medium pH 7.6 + lysine; KE medium pH 5.8 or KE medium pH 5.8 + lysine) supplemented with the respective antibiotics. Bioluminescence and growth were determined every 15 min in the microtiter plates with a Tecan Infinite F500 system (Tecan, Crailsheim, Germany). Data are reported as relative light units (RLU) in counts per second per milliliter per OD_{600}.

In order to analyze signal transduction activity of mNG-CadC *in vivo*, the strains MG1655 wild type or MG1655 mNG-CadC were transformed with the reporter plasmid pBBR1-MCS5-P_{cadBA}-*lux* and treated as described above.

Measurement of intracellular CadA activity. Enzymatic activity of CadA in the *E. coli* strains LF1 and LF1_Δ*cadC_cadC*$_{relocated}$ was determined as described earlier [5,6].

Detection of mCherry-tagged CadC and variants via Western blot. To control protein production of mCherry-tagged CadC and mCherry-tagged CadC variants, *E. coli* MG1655 carrying pET-mCherry-*cadC* or its variants were grown to an OD_{600} of 0.5 in LB supplemented with ampicillin. Overproduction of CadC and its variants was induced by adding 0.5 mM IPTG, and bacteria were harvested 2 h post-induction and then adjusted to OD_{600} = 1. The proteins were fractionated by SDS-PAGE [7] on 12.5% acrylamide gels and transferred to a nitrocellulose membrane. Tagged proteins were labeled with primary polyclonal α-mCherry antibody (ThermoFischer, München, Germany) and the α-rabbit alkaline phosphatase-conjugated antibody (Rockland Immunochemicals, Hamburg, Germany) was used as the secondary antibody according to the manufacturer's recommendations. Localization of the secondary antibody was visualized using colorimetric detection of alkaline phosphatase activity with 5-Bromo-4-chloro-3-indolyl phosphate (BCIP) and nitro blue tetrazolium chloride (NBT). In order to verify the location of mCherry-CadC in different compartements of the cell, cells were disrupted by passage through a high-pressure cell disrupter (Constant Systems, Northants, United Kingdom) in ice-cold disruption buffer (50 mM Tris-HCl pH 7.5, 10 % (v/v) glycerol, 10 mM $MgCl_2$, 100 mM NaCl, 1 mM dithiotreitol, 0.5 mM PMSF and 0.03 mg ml^{-1} DNase). After removal of intact cells and cell debris (= pellet) via centrifugation (9,000 x *g*, 30 min, 4 °C), membrane vesicles were collected by ultracentrifugation at (45,000 x *g*, 60 min, 4 °C), whereas the pellet contained the membrane fraction and the supernatant the cytoplasm. These fractions were separated by SDS-PAGE and processed as described above. As ladder the PageRuler Prestained Protein Ladder (10 to 180 kDa, Thermo Fisher, München, Germany) was used.

Supplementary Figures

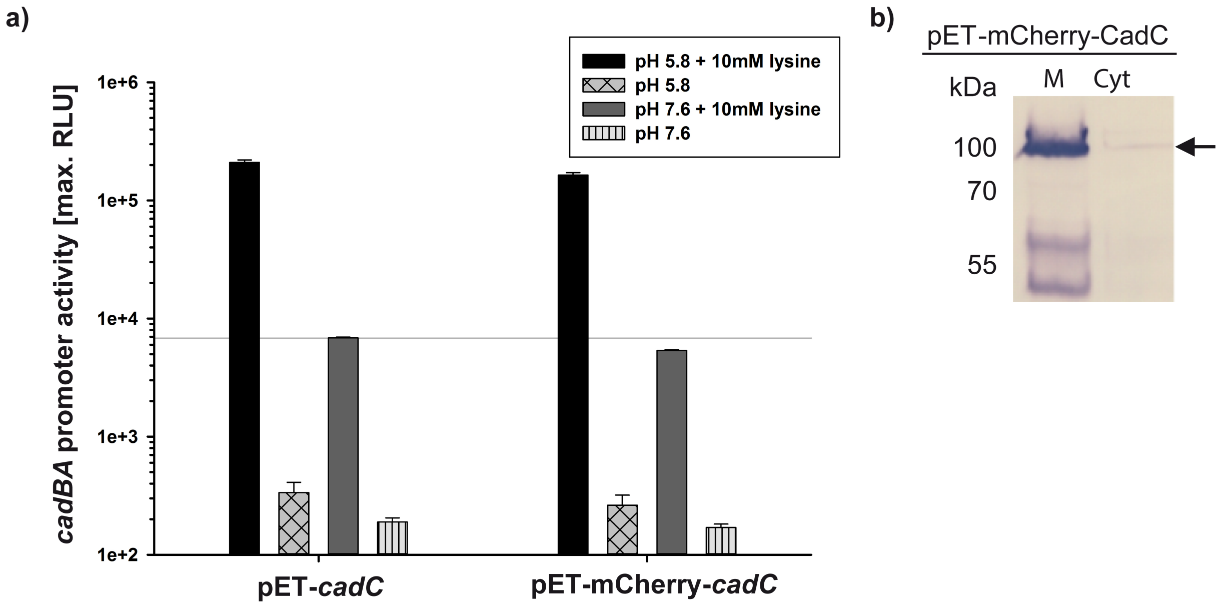

Supplementary Figure 1: mCherry-CadC functions like wild type CadC. (**a**) Functionality of CadC was tested by measuring *cadBA* promoter activity using luminescence as read-out. Assays were performed using *E. coli* MG1655 co-transformed with the reporter plasmids pBBR1-MCS5-P_{cadBA}-*lux* and either pET-*cadC* or pET-mCherry-*cadC* at different conditions. Maximal relative light units (RLU) are shown. The grey line indicates the background level measured for the reporter strain under non-stress conditions (pH 7.6 + lysine: 5,354 RLU). All experiments were performed at least three times, and error bars represent standard deviation of the means. Conditions: pH 5.8 + 10 mM lysine = black bars; pH 5.8 = light grey bars with crossed lines; pH 7.6 + 10 mM lysine = dark grey bars; pH 7.6 = light grey bars with vertical lines. (**b**) Western blot of mCherry-CadC in order to verify production and integration of mCherry-CadC into the cytoplasmic membrane of *E. coli*. *E. coli* MG1655 cells transformed with pET-mCherry-*cadC* was fractionated into membranes and cytoplasm, and mCherry-CadC was detected after SDS-PAGE and Western blotting using α-mCherry antibodies. Black arrow indicates mCherry-CadC full-length protein with a size of 92 kDa. M = membrane fraction, Cyt = cytoplasm.

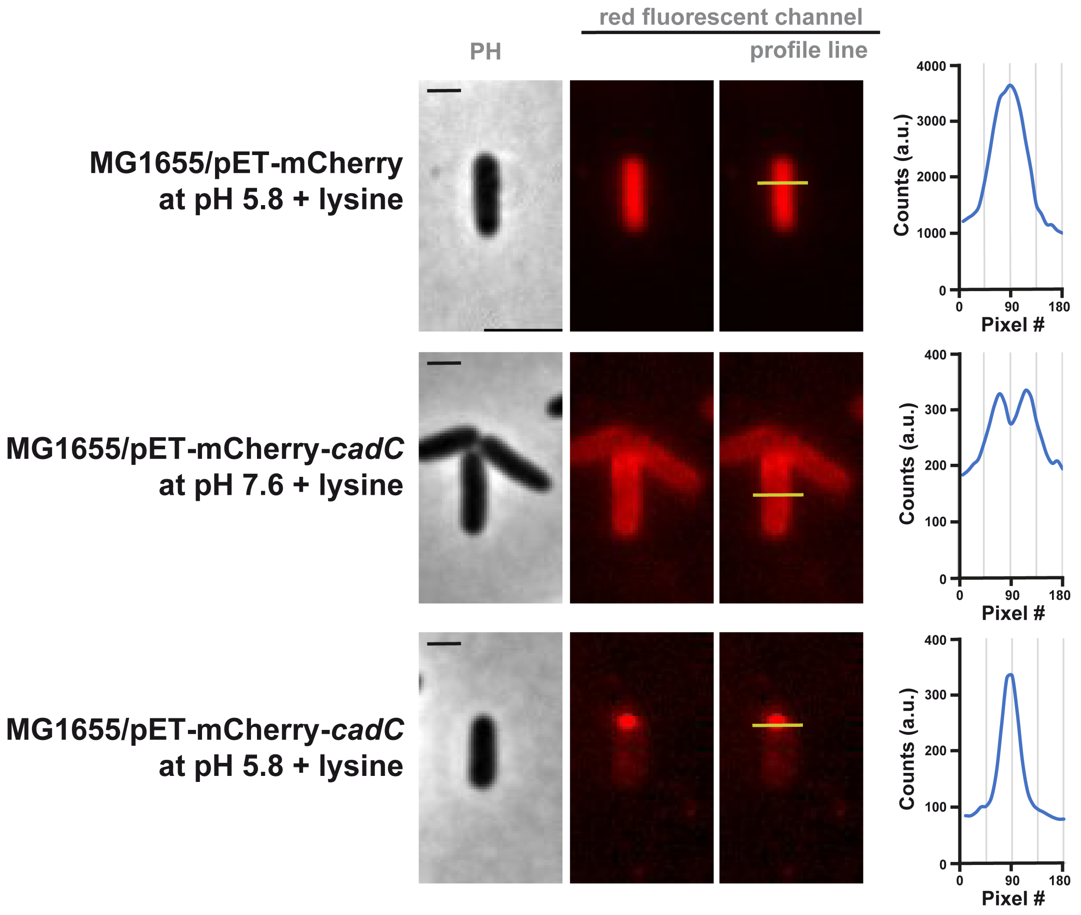

Supplementary Figure 2: Comparison of the intensity profile of mCherry versus mCherry-CadC under stress and non-stress conditions. Graphs on the right side present the intensity profiles of mCherry or mCherry-CadC along the yellow line shown in the red fluorescent images by ImageJ [8]. Microscopy images on the left-hand side illustrate the phase contrast (PH) and red fluorescent channel of the cells. Scale bar = 2µm.

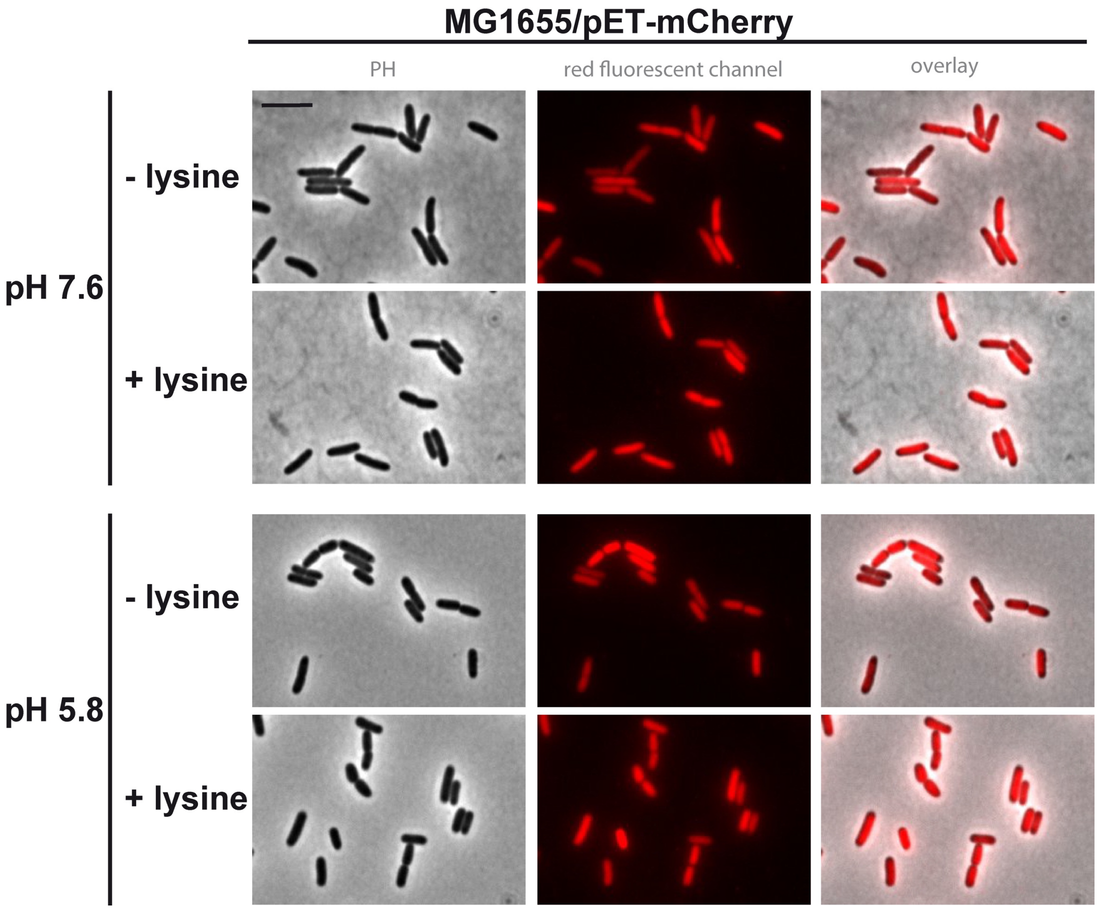

Supplementary Figure 3: Localization of mCherry under stress and non-stress conditions. Fluorescent microscopy images of *E. coli*/pET-mCherry cells grown in minimal medium (glucose as carbon source) buffered at pH 7.6 or pH 5.8, each supplemented with lysine. Images were taken 60 min after exposure to the different conditions. PH = phase contrast, scale bar = 5 µm.

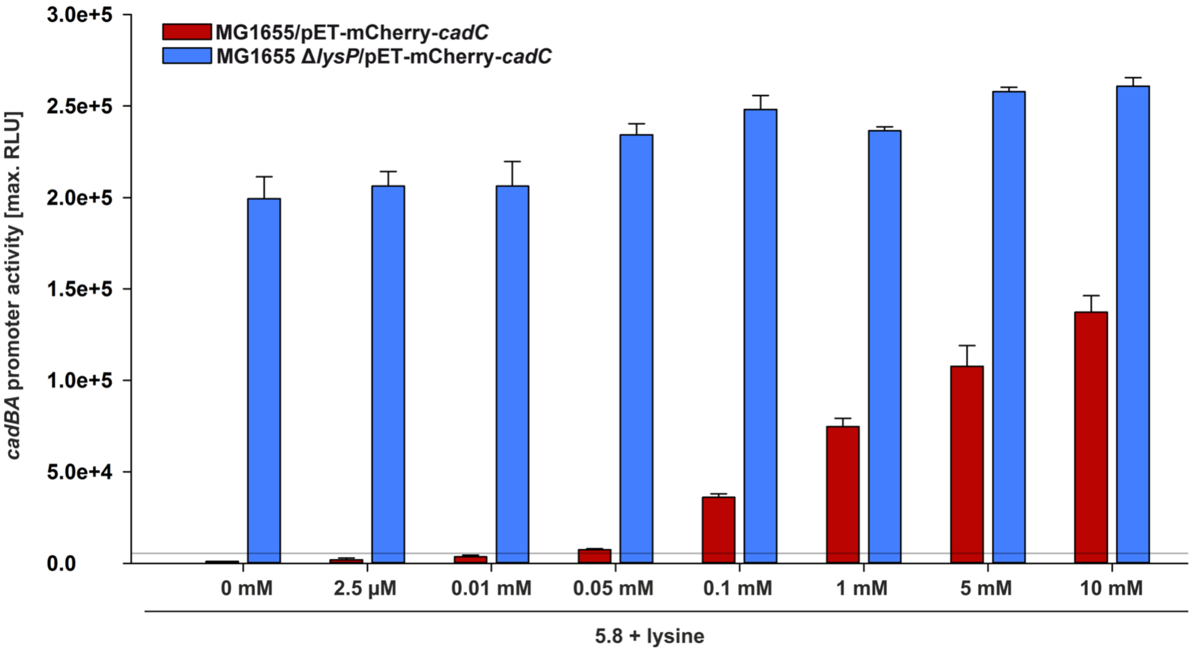

Supplementary Figure 4: Influence of external lysine and the co-sensor LysP on *cadBA* promoter activation. *E. coli* MG1655 wild type and the isogenic *lysP* mutant (each co-transformed with plasmids pBBR1-MCS5-P_{cadBA}-*lux* and pET-mCherry-*cadC)* were grown in medium at pH 5.8 with different external lysine concentrations. The grey line indicates the background level measured for the reporter strain under non-stress conditions (pH 7.6 + lysine: 5,354 RLU). Reporter assays were performed as described in Supplementary Figure 1.

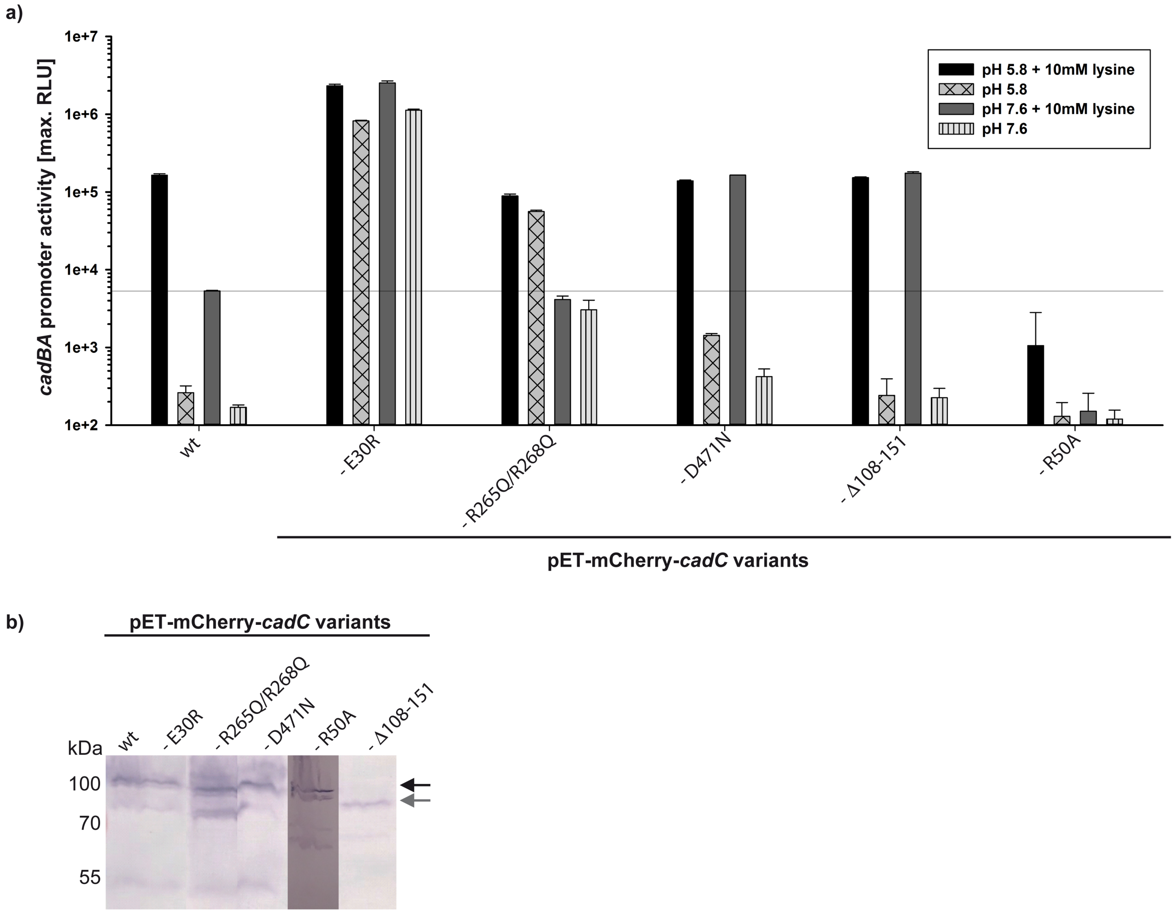

Supplementary Figure 5: Altered stress response of CadC variants. (**a**) Attachment of mCherry did not alter the behavior of the previously characterized CadC variants: CadC-E30R [9], CadC-R265Q/R268Q [10], CadC-D471N [11], CadC-Δ108-151 [12] or CadC-R50A [9]; CadC wild type for comparison. Conditions: pH 5.8 + 10 mM lysine = black bars; pH 5.8 = light grey bars with crossed lines; pH 7.6 + 10 mM lysine = dark grey bars; pH 7.6 = light grey bars with vertical lines. The grey line indicates the background level measured for the reporter strain under non-stress conditions (pH 7.6 + lysine: 5,354 RLU). Reporter assays were performed as described in Supplementary Figure 1. (**b**) Western blot of mCherry-CadC variants in order to verify production and integration of mCherry-CadC into the cytoplasmic membrane of *E. coli*. Arrows indicates mCherry-CadC variants with a size of 92 kDa (black) and mCherry-CadCΔ108-151 with a size of 87 kDa (grey).

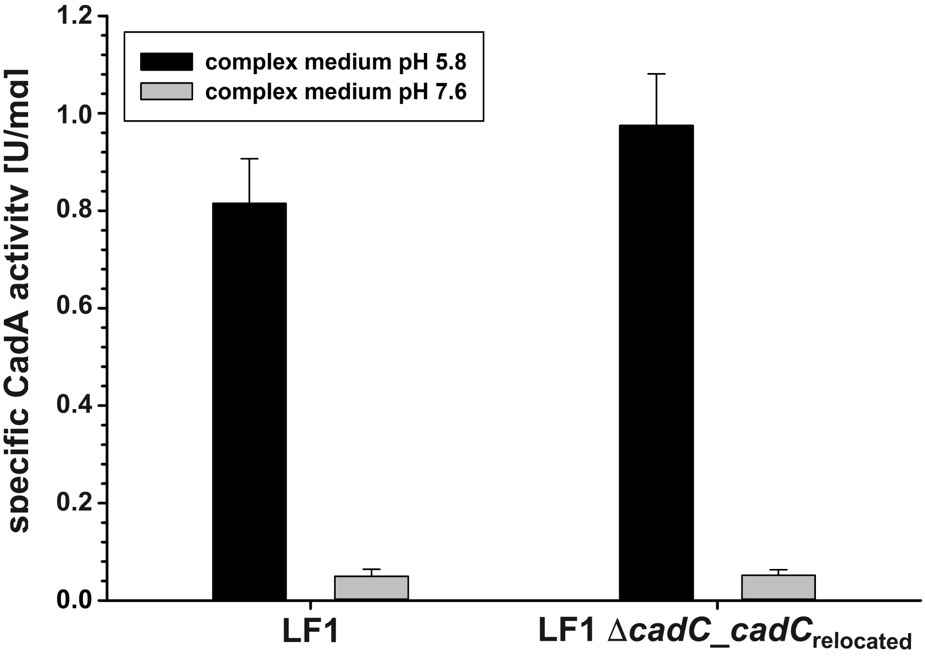

Supplementary Figure 6: Chromosomal re-localization of the *cadC* gene does not affect CadA activity. Specific CadA activity was determined in *E. coli* LF1 and LF1_Δ*cadC*_*cadC*$_{\text{relocated}}$ (relocation of *cadC* within the *lac* operon; native *cadC* is deleted) as described earlier [5,6]. Cells were cultivated in complex medium (LB) either at pH 5.8 (black bars) or pH 7.6 (grey bars).

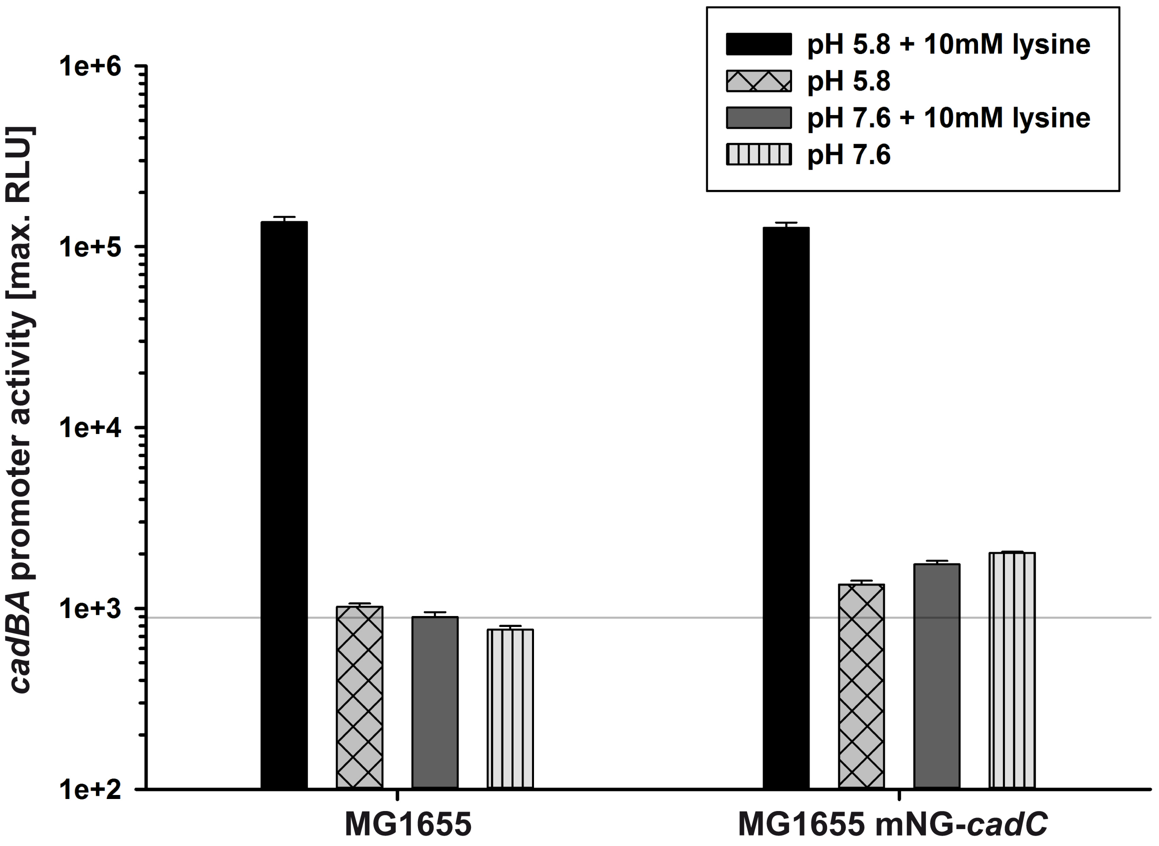

Supplementary Figure 7: Chromosomally integrated mNG-CadC functions like wild type CadC. Reporter assays were performed using *E. coli* strains MG1655 and MG1655 mNG-CadC, each transformed with the reporter plasmid pBBR1-MCS5-P_{cadBA}-*lux*. Conditions: pH 5.8 + 10 mM lysine = black bars; pH 5.8 = light grey bars with crossed lines; pH 7.6 + 10 mM lysine = dark grey bars; pH 7.6 = light grey bars with vertical lines. The grey line indicates the background level measured for the reporter strain under non-stress conditions (pH 7.6 + lysine: 855 RLU). Reporter assays were performed as described in Supplementary Figure 1.

Supplementary Table 1: Summary of percentage of cells with mCherry-CadC cluster(s) in different strains and under various conditions. For each strain or mCherry-CadC variant at least 150 cells per condition were analyzed of three independent experiments. Standard deviation was calculated of the means of the biological triplicates.

	**% of cells with mCherry-CadC cluster(s) at pH 5.8 at various lysine concentrations**					
***E. coli* strain**	**+ 10 mM lysine**	**+ 5 mM lysine**	**+ 1 mM lysine**	**+ 100 µM lysine**	**+ 2.5 µM lysine**	**no lysine**
MG1655	99.3 ± 0.7	92.5 ±1.9	85.9 ± 5.7	9.9 ± 2.2	1.1 ± 0.4	0
MG1655 Δ*lysP*	92.2 ± 5.7	82.2 ± 4.3	63.1 ± 3.2	64.2 ± 1.2	55.9 ± 3.7	70.4 ± 4.9

	**% of cells with mCherry-CadC cluster(s) at pH 5.8 + 10 mM lysine at various cadaverine concentrations**				
***E. coli* strain**	**+ 1 mM cadaverine**	**+ 320 µM cadaverine**	**+ 150 µM cadaverine**	**+ 80 µM cadaverine**	**no cadaverine**
MG1655	0	32.5 ± 3.7	80.6 ± 1.8	97.4 ± 2.3	98.9 ± 0.4

	**% of cells with cluster(s) of CadC-mCherry variants under various conditions**			
**CadC**	**pH 5.8 + 10mM lysine**	**pH 5.8**	**pH 7.6 + 10mM lysine**	**pH 7.6**
CadC	99.3 ± 0.7	0	0.5 ± 0.4	0
CadC-D471N	72.6 ± 1.8	0.2 ± 0.4	80.8 ± 4.0	0
CadC-R265Q/R268Q	81.7 ± 4.7	72.5 ± 3.1	0	0
CadC-Δ108-151	99.1 ± 0.4	2.2 ± 0.4	98.3 ± 0.9	0.9 ± 0.4
CadC-E30R	82.5± 2.0 *	91.9 ± 2.5 *	92.8 ± 3.1 *	92.1 ± 3.5 *
CadC-R50A	8.2 ± 1.7	0.2 ±0.4	0	0

	**% of cells with mCherry-CadC cluster(s) at pH 5.8 + 10 mM lysine under various conditions**
MG1655: 0.4% glycerol	95.4 ± 3.0
MG1655 Δ*PcadBA*	19.3 ± 3.7
MG1655 + pBBR1-P*cadBA*	92.5 ± 4.6 *
MG1655 + P*cadBA*_terminus	98.2 ± 0.4
MG1655 + chloramphenicol	0

* multiple cluster per cell.

Supplementary Table 2: Summary of trajectory numbers of mNeonGreen-CadC during different conditions in MG1655 wild type.

mNeonGreen-CadC	pH 5.8 + 10 mM lysine	pH 7.6
tracks	114	55
movies	24	25
cells	49	32

Supplementary Table 3: Bacterial strains and plasmids used in this study

Strain or plasmids	Relevant genotype or description	Reference or source
**Strains**		
E. coli DH5αλpir	*endA1 hsdR17 glnV44 (= supE44) thi-1 recA1 gyrA96 relA1* φ80'*lac*Δ*(lacZ)*M15 Δ*(lacZYA-argF)*U169 *zdg*-232::Tn10 *uidA::pir+*	[13]
E. coli WM3064	*thrB*1004 *pro thi rpsL hsdS lacZ*ΔM15 RP4-1360 Δ(*araBAD*)567 Δ*dapA*1341::[*erm pir*]	[14]
E. coli MG1655	K-12 F⁻ λ⁻ *ilvG⁻ rfb-50 rph-1*	[15]
E. coli MG1655Δ*lysP*	Clean deletion of *lysP* in MG1655	This work
E. coli MG1655ΔP_{cadBA}	Clean deletion of *cadBA* promoter region in MG1655	This work
E. coli MG1655ΔP_{cadBA} _P_{cadBA}_terminus	Clean deletion of *cadBA* promoter region in MG1655 with relocated *cadBA* promoter region at the terminus	This work
E. coli MG1655 mNG-*cadC*	Clean insertion of N-terminal tagged *cadC* with *mNeonGreen* (mNG) in MG1655	This work
LF1	MG1655 *rpsL*150 P_{lac}::*rpsL-neo-kan::lacZ*$^{Δ1–100\ bp}$; Kan^R $Strp^S$	[16]
LF1_Δ*cadC* _*cadC*relocated	MG1655 *rpsL*150 P_{cadC}-*cadC*::*lacZ*, Kan^S,	[6]
**Plasmids**		
pET16b	Overexpression plasmid for His-tagged proteins, Amp^R	Novagen
pET16b-*cadC*	N-terminal 10His-tagged *cadC* in pET16b, Amp^R	[17]
pET-mCherry-*cadC*	N-terminal fusion of CadC with mCherry, connected with a 22 aa long linker containing a 10His tag in pET16b, Amp^R	This work
pET-*cadC*-D471N	*cadC*-D471N in pET16b, Amp^R	[11]
pET-mCherry-*cadC*-D471N	*cadC*-D471N tagged N-terminal with mCherry in pET16b, Amp^R	This work
pET-*cadC*-E30R	*cadC*-E30R in pET16b, Amp^R	[9]
pET-mCherry-*cadC*-E30R	*cadC*-E30R tagged N-terminal with mCherry in pET16b, Amp^R	This work
pET-*cadC*-Δ108-151	*cadC*-Δ108-151 (deletion of amino acids 108-151) in pET16b, Amp^R	[12]
pET-mCherry-*cadC*-Δ108-151	*cadC*-Δ108-151 tagged N-terminal with mCherry in pET16b, Amp^R	This work
pET-*cadC*-R265Q/R268Q	*cadC*-R265Q/R268Q in pET16b, Amp^R	[10]
pET-mCherry-*cadC*-R265Q/R268Q	*cadC*-R265Q/R268Q tagged N-terminal with mCherry in pET16b, Amp^R	This work

pET-*cadC*-R50A	*cadC*-R50A in pET16b, Amp^R	[9]
pET-mCherry-*cadC*-R50A	*cadC*-R50A tagged N-terminal with mCherry in pET16b, Amp^R	This work
pBR322Cherry	mCherry in pBR322, Amp^R	[2]
pET-mCherry	mCherry in pET16b, Amp^R	This work
pBBR1-MCS5-TT-RBS-*lux*	*luxCDABE* and terminators lambda *T0 rrnB1 T1* cloned into pBBR1-MCS5 for plasmid-based transcriptional fusions, Gm^R	[3]
pBBR1-MCS5-P$_{cadBA}$-*lux*	*cadBA* promoter controlling expression of *luxCDABE*, in pBBR1-MCS5-TT-RBS-*lux*, Gm^R	This work
pNTPS138-R6KT	*mobRP4*$^+$ *ori*-R6K *sacB*; suicide plasmid for in-frame deletions, Km^R	[18]
pNTPS138-R6KT-Δ*lysP*	pNPTS-138-R6KT-derived suicide plasmid for in-frame deletion of *lysP* in MG1655, Km^R	This work
pNTPS138-R6KT-*mNG-cadC*	pNPTS-138-R6KT-derived suicide plasmid for in-frame insertion of *mNeonGreen* (mNG) in front of *cadC* in MG1655 strains, Km^R	This work
pNTPS138-R6KT-P$_{cadBA}$_terminus	pNPTS-138-R6KT-derived suicide plasmid for insertion of *cadBA* promoter region at terminus in MG1655ΔP$_{cadBA}$ strain, Km^R	This work

References

1. Gibson, D. G. *et al.* Enzymatic assembly of DNA molecules up to several hundred kilobases. *Nat. Meth.* **6,** 343–345 (2009).
2. Munch, A., Stingl, L., Jung, K. & Heermann, R. *Photorhabdus luminescens* genes induced upon insect infection. *BMC Genomics* **9,** 229 (2008).
3. Gödeke, J., Heun, M., Bubendorfer, S., Paul, K. & Thormann, K. M. Roles of two *Shewanella oneidensis* MR-1 extracellular endonucleases. *Appl. Environ. Microbiol.* **77,** 5342–5351 (2011).
4. Schenk, K. *et al.* Rapid turnover of DnaA at replication origin regions contributes to initiation control of DNA replication. *PLoS Genet* **13,** e1006561–32 (2017).
5. Fritz, G. *et al.* Induction kinetics of a conditional pH stress response system in *Escherichia coli*. *J. Mol. Biol.* **393,** 272–286 (2009).

6. Buchner, S. G. Strukturelle und funktionale Analyse der Effektordomäne des pH-abhängigen Einkomponentensystems CadC in *Escherichia coli*. *Dissertation LMU* 1–136 (2015).

7. Laemmli, U. K. Cleavage of structural proteins during the assembly of the head of bacteriophage T4. *Nature* **227,** 680–685 (1970).

8. Schneider, C. A., Rasband, W. S. & Eliceiri, K. W. NIH Image to ImageJ: 25 years of image analysis. *Nat. Meth.* **9,** 671–675 (2012).

9. Schlundt, A. *et al.* Structure-function analysis of the DNA-binding domain of a transmembrane transcriptional activator. *Sci. Rep.* **7,** 1–16 (2017).

10. Rauschmeier, M., Schüppel, V., Tetsch, L. & Jung, K. New insights into the interplay between the lysine transporter LysP and the pH sensor CadC in *Escherichia coli*. *J. Mol. Biol.* **426,** 215–229 (2014).

11. Haneburger, I., Eichinger, A., Skerra, A. & Jung, K. New insights into the signaling mechanism of the pH-responsive, membrane-integrated transcriptional activator CadC of *Escherichia coli*. *J. Biol. Chem.* **286,** 10681–10689 (2011).

12. Buchner, S., Schlundt, A., Lassak, J., Sattler, M. & Jung, K. Structural and functional analysis of the signal transducing linker in the pH-responsive one component system CadC of *Escherichia coli*. *J. Mol. Biol.* **457,** 2548–2561 (2015).

13. Miller, V. L. & Mekalanos, J. J. A novel suicide vector and its use in construction of insertion mutations: osmoregulation of outer membrane proteins and virulence determinants in *Vibrio cholerae* requires *toxR*. *J. Bacteriol.* **170,** 2575–2583 (1988).

14. Dehio, C. & Meyer, M. Maintenance of broad-host-range incompatibility group P and group Q plasmids and transposition of Tn5 in *Bartonella henselae* following conjugal plasmid transfer from *Escherichia coli*. *J. Bacteriol.* **179,** 538–540 (1997).

15. Blattner, F. R. *et al.* The complete genome sequence of *Escherichia coli* K-12. *Science* **277,** 1453–1462 (1997).

16. Fried, L., Lassak, J. & Jung, K. A comprehensive toolbox for the rapid construction of *lacZ* fusion reporters. *J. Microbiol. Methods* **91,** 537–543 (2012).

17. Küper, C. & Jung, K. CadC-mediated activation of the *cadBA* promoter in *Escherichia coli*. *J. Mol. Microbiol. Biotechnol.* **10,** 26–39 (2005).

18. Lassak, J., Henche, A.-L., Binnenkade, L. & Thormann, K. M. ArcS, the cognate sensor kinase in an atypical Arc system of *Shewanella oneidensis* MR-1. *Appl. Environ. Microbiol.* **76,** 3263–3274 (2010).
