# Supplementary material for: DNA-binding directs the localization of a membrane-integrated receptor of the ToxR family
Source: Commun Biol. 2019 Jan 4;2:4. doi: 10.1038/s42003-018-0248-7 (PMC6320335; doi:10.1038/s42003-018-0248-7)
Supplement: Supplementary file 2 — Description of Additional Supplementary Files [file 42003_2018_248_MOESM2_ESM.docx]

**Description of Additional Supplementary Files**

**File Name**: Supplementary Data 1

**Description**: Primers used in this study.
